# A Simple Yet Novel Way to Use a Catheter

**Published:** 1881-08

**Authors:** Edward C. Huse

**Affiliations:** Rockford, Illinois


### ARTICLE VIII.

### A SIMPLE YET NOVEL WAY TO USE A CATHETER. By EDWARD C. HUSE, M.D., Rockford, Illinois.

The soft rubber catheters of Jacques and Nélaton, are a wondrous improvement over the old-fashioned urethral "spike" style of instrument. Doubtless hundreds of lives have already been saved by them which would have been sacrificed, as formerly, by the old barbarity.

We are all familiar with the bundle of one-eyed "spike" catheters, of varying sizes, in the show-case of every druggist, which, in fact, probably always will be seen there. Most of them appear to have been "in stock" a hundred years.

A device for using the soft rubber instrument to advantage, in a narrowed urethra was brought forward by Tiemann & Co., some time since, acting upon the suggestion of a well known professor in New York. The very absence of rigidity in the soft instrument, excludes its use in prostatic enlargement, if complicated with stricture. The "spike" instrument is of course highly objectionable unless the wire stylet be withdrawn, when it becomes, virtually, no better than the very soft ones.

The new design for stylet which Tiemann & Co. make, to be used on occasion, is undoubtedly an excellent thing. But if one will use the "spike," and, when it reaches the bulbo-membranous region (where it is apt to stop), will simply *withdraw* the stylet for an inch or a inch and a half, he will find substantially the same instrument in his hands as the new device, and that the rigid shaft, coupled with the soft, persuasive extremity are thus in his hands in a moment.

A case in point will illustrate this. Some two months since, O. B. S., aged 22, with a deep seated and tight stricture of three years standing, came to me for treatment. After dilating carefully for several weeks with *bougies à boule*, till a No. 6 could be got through, I resolved to use the *one-sitting* treatment, and accordingly operated with the divulsor of Thompson. There had been a prostatic abscess for somewhat over a year, the result, he told me, of small and sharp bougies, Nos. 3 and 4, which he had *used upon himself*, after " reading up " on the subject. Some eight hours after the operation I withdrew a No. 12 bougie, which was introduced immediately after the divulsor had done its work, and attempted, by means of a " velvet eyed " soft catheter, to empty the bladder. It did not pass. The catheter, No. 12, was worked along, down to the prostatic region, and there stopped.

An experience of several thousand times passing the rigid silver instrument has shown me, as it ought to every one, that no *force* in such a case is ever admissible, or for that matter in *any* case where a catheter is used. *En passant*, how often this vital, or rather, mortal, principle is ignored! What a hecatomb of human sacrifice its disregard has cost! The vertebrated instrument of Squire did no better. What was needed was a rigid shaft with a *coaxing* end. Taking a No. 12 "spike," I went down carefully to the obstruction, withdrew, for an inch or so, the stylet, thus leaving a *climbing* or *roving* distal end to the instrument, pressed gently upon the shaft, and in a few moments a warm jet of urine showed me that the prostatic Rubicon was passed.

It is not easy, always, for country practitioners to get " velvet-eyed " or soft catheters, with complicated stylets and more complicated instructions. Especially is this true in winter, where muddy roads, ten foot snow banks or —40° place an embargo on the fulfillment of one's intention. It is just at this season that prostatic troubles are worst. For fifteen cents, and the simplest possible manipulation, many an accident, usually fatal, could have been and can be now avoided.

It is not my purpose, in conclusion, to claim anything startling or especially ingenious, in this article. It is simply a new adaptation of an old means to the fulfillment of a very important purpose, and thus the avoidance of a sunken rock, in our surgical navigation on which the life of many a patient and the reputation of many a physician, have been irretrievably stranded.

June 12, 1881.

